# Supplementary material for: BART3D: inferring transcriptional regulators associated with differential chromatin interactions from Hi-C data
Source: Bioinformatics. 2021 Mar 15;37(18):3075–8. doi: 10.1093/bioinformatics/btab173 (PMC8479658; doi:10.1093/bioinformatics/btab173)
Supplement: btab173_Supplementary_Data [file btab173_supplementary_data.pdf]

SUPPLEMENTARY DATA

BART3D: Inferring transcriptional regulators associated with differential chromatin interactions from Hi-C data

Zhenjia Wang, Yifan Zhang, Chongzhi Zang

Supplementary data include Supplementary methods, 5 Supplementary figures, and 6 Supplementary tables.

Supplementary Figure S1. Hi-C read count negatively correlates with genomic distance between bin pairs.

Supplementary Figure S2. Effect of normalization.

Supplementary Figure S3. Comparison of different normalization methods.

Supplementary Figure S4. Comparison between DCI and chromatin loops.

Supplementary Figure S5. BART3D results on dynamic Hi-C datasets with TR perturbation under different genomic distance settings.

Supplementary Table S1. List of Hi-C data used in this work.

Supplementary Table S2. List of HiChIP data used in this work.

Supplementary Table S3. List of ChIP-seq data used in this work.

Supplementary Table S4. Comparison of BART3D results between individual replicates and replicate combined data.

Supplementary Table S5. Optional adjustment of DCI by locus read coverage.

Supplementary Table S6. Comparison of BART3D TR prediction with Selfish and diffHiC.

SUPPLEMENTARY METHODS

Data collection

Hi-C, HiChIP, and ChIP-seq data were collected from NCBI GEO (Barrett *et al.*, 2013) in fastq format. Detailed information including accession numbers of all samples used in this work can be found in Supplementary Tables S1-S3.

Data processing

Hi-C (Supplementary Table S1) and HiChIP (Supplementary Table S2) sequence reads were aligned to the human (hg38) or mouse (mm10) reference genomes and processed using HiC-Pro (Servant *et al.*, 2015). Contact matrices were generated at a resolution of 5kb and normalized as described in *Normalization of contact matrices*. ChIP-seq (Supplementary Table S3) reads were aligned to the mouse reference genome (mm10) using BWA (Li and Durbin, 2009). Sam files were then converted into bam files using samtools (Li *et al.*, 2009). MACS2 (Zhang *et al.*, 2008) was used to call peaks under the FDR threshold of 0.05.

Normalization of contact matrices

Given a Hi-C contact matrix $A = \{a_{ij}\}$, the observed read count a_{ij} represents the interaction frequency between a pair of genomic bins i and j. To account for the negative correlation between the intra-chromosomal interaction frequency and the genomic distance between the bin pair (Lieberman-Aiden *et al.*, 2009), we normalized the contact matrix of each chromosome as follows: for any given genomic distance $d_k = k * r$, where r is the bin size (data resolution), we employed a normalization factor $\bar{S}_{d_k}$ as the average read count across all bin pairs with the same genomic distance d_k in this chromosome, i.e., $\bar{S}_{d_k} = (\sum_{j-i=k} a_{ij})/n$, where *n* is the total number of bin pairs with distance d_k. The read count a_{ij} of the bin pair with distance d_k was

normalized by $\bar{S}_{d_k}$ as $a'_{ij} = a_{ij}/\bar{S}_{d_k}$. The matrix A was normalized into $A' = \{a'_{ij}\}$ for each chromosome.

Detection of differential chromatin interactions

Considering *m* Hi-C matrices for treatment and *n* Hi-C matrices for control (m,n≥1) as input, we denoted the normalized matrix $T^i = \{t^i\}$ as the *i*-th treatment matrix and $C^j = \{c^j\}$ as the *j*-th control matrix, (*i*=1, …, *m*; *j*=1, …, *n*). $\mathcal{B} = \{1, 2, \dots, \lfloor l/r \rfloor\}$ represents all equal-sized non-overlapping bins within a chromosome, where l is the length of the chromosome and r is the bin size. For a given genomic region $x \in \mathcal{B}$ and a pre-defined range of genomic distance L, the interaction frequencies between x and its flanking regions with genomic distance up to L were collected, as $IT^i = \{t^i_{xk}\}$ from T and $IC^j = \{c^j_{xk}\}$ from C, respectively, where $k \in \mathcal{B}, x - L/r \leq k \leq x + L/r$. The t-statistic at x was calculated using the paired-sample *t*-test between the two arrays of interaction frequencies IT^i and IC^j as follows:

$$d_{xk} = t^i_{xk} - c^j_{xk},$$

$$t^{ij} = \frac{\bar{d}}{s_d/\sqrt{n}}$$

where d_{xk} is the difference in interactions between each paired element in IT^i and IC^j; $\bar{d}$ and s_d are the mean and standard deviation of $\{d_{xk}\}$, respectively; n is the length of each array and $n = 2L/r + 1$. The estimated p-value of t^{ij} is p^{ij}.

Assume we have k^+ p-values estimated from positive t-statistics and k^- p-values estimated from negative t-statistics, $k^+ + k^- = mn$. We use Fisher's method (Fisher, 1925) to combine all the k^+ p-values as:

$$S = -2\sum_{i=1}^{k^+} \ln{(p^i)}$$

The statistic S follows a Chi-square distribution with $2k^+$ degrees of freedom. Under this statistical distribution, a p-value can be determined as p^+ to quantify the significance of chromatin interaction increases between multiple treatment and control matrices at the genomic region x. Meanwhile, the k^- p-values estimated from negative t-statistic can be combined as p^- using the same approach. The differential chromatin interaction (DCI) score at the given genomic region can be calculated using the logarithm of p^+ or p^- as follows:

$$DCI = \begin{cases} -\log_{10}(p^+), if\ k^+ > k^-, or\ k^+ = k^-;\ p^+ < p^- \\ \log_{10}(p^-), if\ k^+ < k^-, or\ k^+ = k^-;\ p^+ > p^- \end{cases}$$

Inference of TRs associated with differential chromatin interactions

We used previously curated union DNaseI hypersensitive sites (UDHS), which include 2,723,010 unique non-overlapping DNase-seq peaks for human and 1,529,448 for mouse, to represent all putative cis-regulatory elements (CREs) in the genome (Wang *et al.*, 2016). A genome-wide DCI profile was generated by calculating the DCI score of every bin across each chromosome. The DCI profile was mapped to UDHS such that the score for each candidate CRE is set to be equal to the DCI score of the genomic bin where the CRE is located.

We used the BART algorithm (Wang *et al.*, 2018) to infer TRs associated with differential chromatin interactions. The analysis was done twice, for inferring TRs associated with increased and decreased chromatin interactions, separately. For increased chromatin interactions, we ranked all CREs decreasingly by their scores, i.e., CREs with high positive scores would be ranked at the front. We calculated an association score between the CRE profile and each TR binding profile for all ChIP-seq datasets. The association score is defined as the area under the ROC curve (AUC) using the DCI score on CRE as the predictor for TR binding, set as a binary value indicating whether the CRE is overlapped with a peak of that TR from the ChIP-seq dataset. To account for multiple ChIP-seq datasets for the same TR, the Wilcoxon rank-sum test

was then applied to assess each TR's significance by comparing the association scores from all ChIP-seq data for this TR with those from all other ChIP-seq datasets, and a background model was used to detect the specificity of each TR. A series of quantification scores with statistical assessments were included for a final ranked list of inferred TRs. For decreased chromatin interactions, the CRE profile was flipped, so that the CREs with the most decreased chromatin interactions are ranked at the front, and the BART analysis was then performed in the same way.

Comparison of BART3D results between using each replicate and using all replicates

For Hi-C data with replicates (Rad21 KO, Smchd1 KO and Srf over expression), we ran BART3D on each treatment replicate vs. each control replicate as well as the replicate combined data to check the robustness of the BART3D algorithm. For example, for Rad21 KO with two replicates in treatment and control, five results were generated as Treatment-rep1 vs. Control-rep1 (T1-C1), Treatment-rep1 vs. Control-rep2 (T1-C2), Treatment-rep2 vs. Control-rep1 (T2-C1), Treatment-rep2 vs. Control-rep2 (T2-C2), and Treatment-all-replicates vs. Control-all-replicates (combined). The Pearson correlation coefficient was calculated between each pair of DCI profiles from BART3D results (Supplementary Table S4a-c). Rank of the relevant TR in each BART3D result were shown in Supplementary Table S4d-f. This direct comparison suggests that the BART3D algorithm generates consistent results from individual replicate and from including all replicates.

Adjustment of DCI profile by locus read coverage difference

Differential locus read coverage between the dynamic Hi-C datasets was calculated as follows: Given *m* treatment Hi-C matrices and *n* control Hi-C matrices ($m,n \geqslant 1$), the differential locus read coverage for a genomic bin x was calculated as:

$$LFC_x = log_2\left(\frac{\bar{t}_x}{\bar{c}_x}\right)$$

where $\bar{t}_x$ and $\bar{c}_x$ are the normalized locus read coverage (Hi-C read count in genomic bin x divided by the average read count across all genomic bins) averaged across all treatment samples or all control samples, respectively. Pearson correlation was calculated between LFC and DCI in each sample as shown in Supplementary Table S5a. As they show weak positive correlations, a linear regression model was applied between DCI and LFC to reduce the effect of LFC in DCI as a confounding factor. The regression model is:

$$DCI \sim a * LFC + b$$

The residual DCI^r was then used as the adjusted DCI for each genomic region:

$$DCI^r = DCI - \widehat{DCI}$$

where $\widehat{DCI}$ is the fitted value from the regression model. BART TR analyses using the original DCI profile and the adjusted DCI profile were performed and the ranks of relevant TRs were compared and shown in Supplementary Table S5b-g. The TR results are overall consistent as the relevant TRs are consistently ranked high. This option of DCI adjustment of is provided in the BART3D package.

Comparison of normalization methods in detecting chromatin interactions

To evaluate the feasibility and performance of Hi-C normalization approaches, we used HiChIP data and tested how different normalization methods affect the inference of the HiChIP target factor. By targeting a specific factor of interest, HiChIP signals are enriched at the target-bound loci (Mumbach *et al.*, 2016). We collected 84 HiChIP datasets targeting different TRs (Supplementary Table S2). For each HiChIP dataset, we generated genomic contact maps at 5kb resolution without normalization, with ICE normalization (Servant *et al.*, 2015), and with distance-based normalization. We then generated a genomic profile from each contact map, in which each 5kb bin across the genome is scored as the sum of interaction signals between this

bin and all of its flanking bins within 500kb. We used BART to infer TRs associated with this genomic interaction profile. We expected that a contact map with appropriate normalization should yield to a BART result in which the HiChIP target factor ranked higher (with higher significance). As a control, we run BART analysis on the HiChIP sequence read pile-up profile and used the rank of the target factor as a reference (Supplementary Fig. S3).

Comparison of BART3D with other methods

BART3D, Selfish (v1.10.2) (Ardakany *et al.*, 2019) and diffHic (v1.14.0) (Lun and Smyth, 2015) were compared on Hi-C data of Ctcf KO, Rad21 KO and Smchd1 KO, in which the KO factors were known as the ground truths. Differential chromatin loops were identified for each Hi-C dataset using Selfish or diffHic with default parameters. Method comparison was performed in 2 aspects: 1) Overlap of identified genomic regions with differential chromatin interaction; and 2) Rank of perturbed TR in the TR prediction result. For differential region comparison, genomic regions with increased interaction (DCI>2) identified by BART3D were compared with anchors of loops with increased interaction (logFC>1, FDR<0.05) identified by Selfish or diffHic. Regions with decreased interaction (DCI<-2) identified by BART3D were compared with anchors of loops with decreased chromatin interaction (logFC<-1, FDR<0.05) identified by Selfish or diffHic (Supplementary Fig. S4). For TR prediction from differential chromatin loops identified from other methods, BART was applied on the increased or decreased loop anchor regions separately. The rank of the relevant TR for each Hi-C dataset was shown in Supplementary Table S6. It is worth noting that genomic regions with differential chromatin interactions defined in BART3D is conceptually different from chromatin loops (Supplementary Fig. S4a,b), the different performance of BART3D from other methods is not unexpected.

Determination of default genomic distance parameter

Using different genomic distance range parameters might lead to different TR inference results, because the acting range of different TRs vary a lot. In practice, users may try different distance parameters for exploratory studies. To set an appropriate default value for this parameter, we applied BART3D on a series of Hi-C datasets (Supplementary Table S1) comparing the wide type with perturbation (deletion or activation) of different TRs using different genomic distance ranges, i.e., 50kb, 100kb, 200kb, 500kb, and 1000kb. We compared the rank of the perturbation target factor in the BART3D results across different genomic distance ranges, and found that 200kb is where most perturbation factors were ranked on top (Supplementary Fig. S5). Therefore, we set 200 kb as the default value for the genomic distance range parameter.

REFERENCES

Ardakany,A.R. *et al.* (2019) Selfish: discovery of differential chromatin interactions via a self-similarity measure. *Bioinformatics*, **35**, i145–i153.

Barrett,T. *et al.* (2013) NCBI GEO: archive for functional genomics data sets—update. *Nucleic Acids Res*, **41**, D991–D995.

Fisher,R.A. (1925) Statistical Methods for Research Workers. In, *undefined*. Oliver and Boyd (Edinburgh).

Li,H. *et al.* (2009) The Sequence Alignment/Map format and SAMtools. *Bioinformatics*, **25**, 2078–2079.

Li,H. and Durbin,R. (2009) Fast and accurate short read alignment with Burrows–Wheeler transform. *Bioinformatics*, **25**, 1754–1760.

Lieberman-Aiden,E. *et al.* (2009) Comprehensive Mapping of Long-Range Interactions Reveals Folding Principles of the Human Genome. *Science*, **326**, 289–293.

Lun,A.T.L. and Smyth,G.K. (2015) diffHic: a Bioconductor package to detect differential genomic interactions in Hi-C data. *Bmc Bioinformatics*, **16**, 258.

Mumbach,M.R. *et al.* (2016) HiChIP: efficient and sensitive analysis of protein-directed genome architecture. *Nat Methods*, **13**, 919–922.

Servant,N. *et al.* (2015) HiC-Pro: an optimized and flexible pipeline for Hi-C data processing. *Genome Biol*, **16**, 259.

Wang,S. *et al.* (2016) Modeling cis-regulation with a compendium of genome-wide histone H3K27ac profiles. *Genome Res*, **26**, 1417–1429.

Wang,Z. *et al.* (2018) BART: a transcription factor prediction tool with query gene sets or epigenomic profiles. *Bioinformatics*, **34**, 2867–2869.

Zhang,Y. *et al.* (2008) Model-based Analysis of ChIP-Seq (MACS). *Genome Biol*, **9**, R137.

Supplementary Figure S1

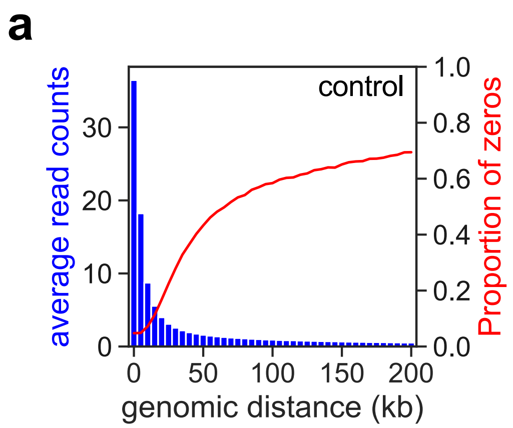

Supplementary Figure S1. Hi-C read count negatively correlates with genomic distance between bin pairs. Average read counts and percentage of zeros in all bin pairs at the same genomic distance in two Hi-C datasets. (a) control: GSM2790405; (b) treatment: GSM2790406.

Supplementary Figure S2

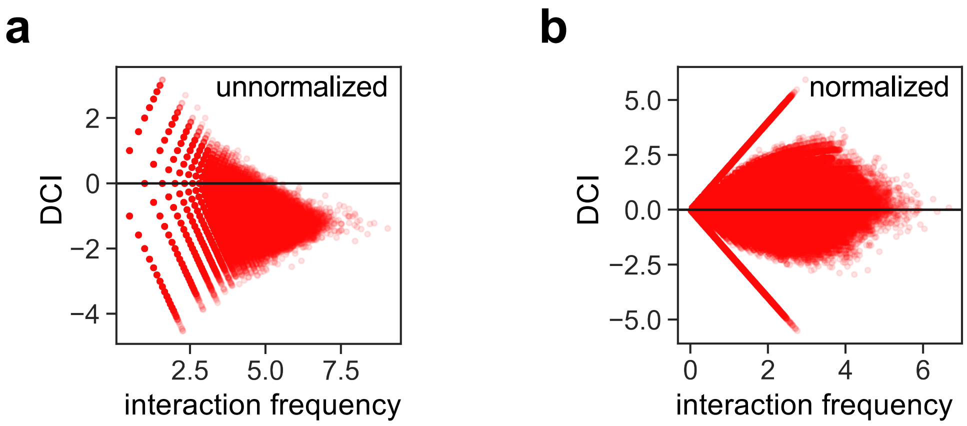

Supplementary Figure S2. Effect of normalization. MA plots of averaged interaction frequency (x-axis) and differential chromatin interaction (DCI, y-axis) with unnormalized (a) and normalized (b) Hi-C contact matrices between treatment (GSM2790406) and control (GSM2790405).

Supplementary Figure S3

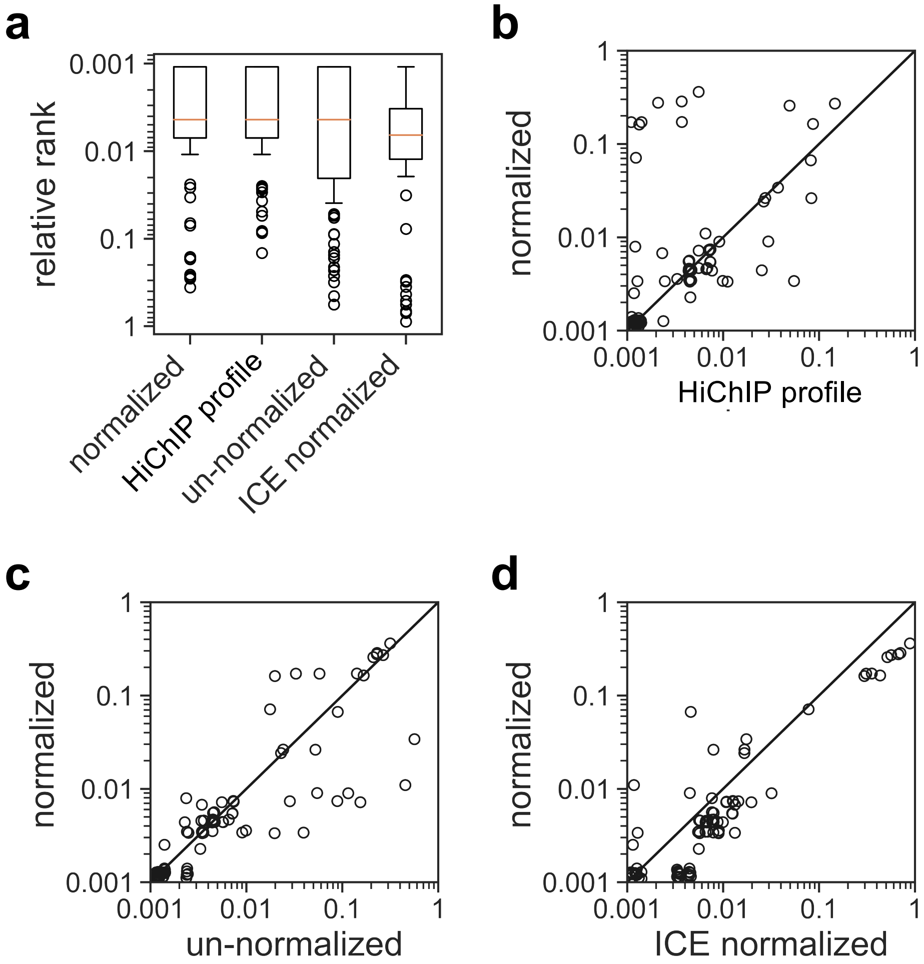

Supplementary Figure S3. Comparison of different normalization methods.

(**a**) BART results of target TRs from 84 HiChIP datasets using different normalization methods. For distance-based normalized (labeled as "normalized"), unnormalized, and ICE normalized, BART was applied to a genomic region profile scored by summarizing the interaction frequencies of each 5kb bin to its flanking bins within 500kb. For the HiChIP profile, BART was applied to the HiChIP sequence read bam file (as positive control). Relative rank represents the rank of the target TR divided by the total number of TRs in the BART library. Center line in the box represents median.

(**b-d**) Comparison of the relative ranks of target TRs in the BART results generated from distance-based normalization against other methods: (**b**) HiChIP profile, (**c**) unnormalized and (**d**) ICE normalization. Each dot represents a dataset from the 84 HiChIP samples. More dots located below the diagonal line indicates that distance-based normalization (y-axis) yields to higher rank in the BART result.

Supplementary Figure S4

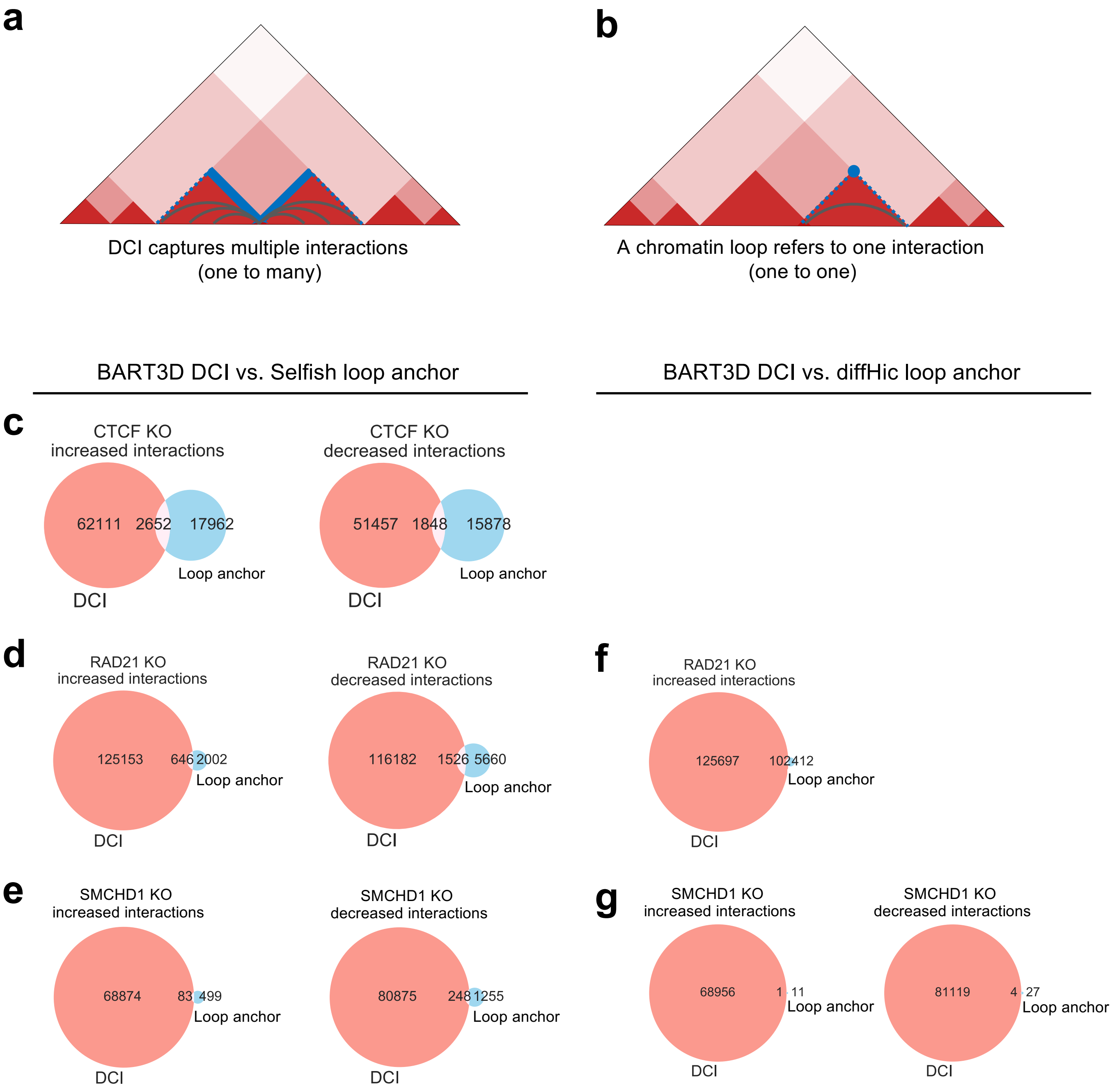

Supplementary Figure S4. Comparison between DCI and chromatin loops. (a) DCI captures multiple chromatin interaction events between one genomic locus and its surrounding loci (arcs), reflected as two solid blue stripes on the Hi-C contact map. **(b)** A chromatin loop refers to one interaction between two loci (arc), reflected as the blue dot on the Hi-C contact map. **(c-e)** Identified regions with increased DCI (DCI>2) and anchors of increased loops (left Venn diagrams), and regions with decreased DCI (DCI<-2) and anchors of decreased loops (right Venn diagrams), comparing BART3D and Selfish using Hi-C data of Ctcf KO **(c)**, Rad21 KO **(d)** and Smchd1 KO **(e)**. $P < 0.05$, by fisher's exact test, in all comparisons. **(f,g)** The same analysis as in (c-e) using the differential loops identified by diffHiC from Hi-C data of Rad21 KO **(f)**, Smchd1 KO **(g)**. N.S., by fisher's exact test, in all comparisons. Panels left blank indicate no differential loop identified using diffHic.

Supplementary Figure S5

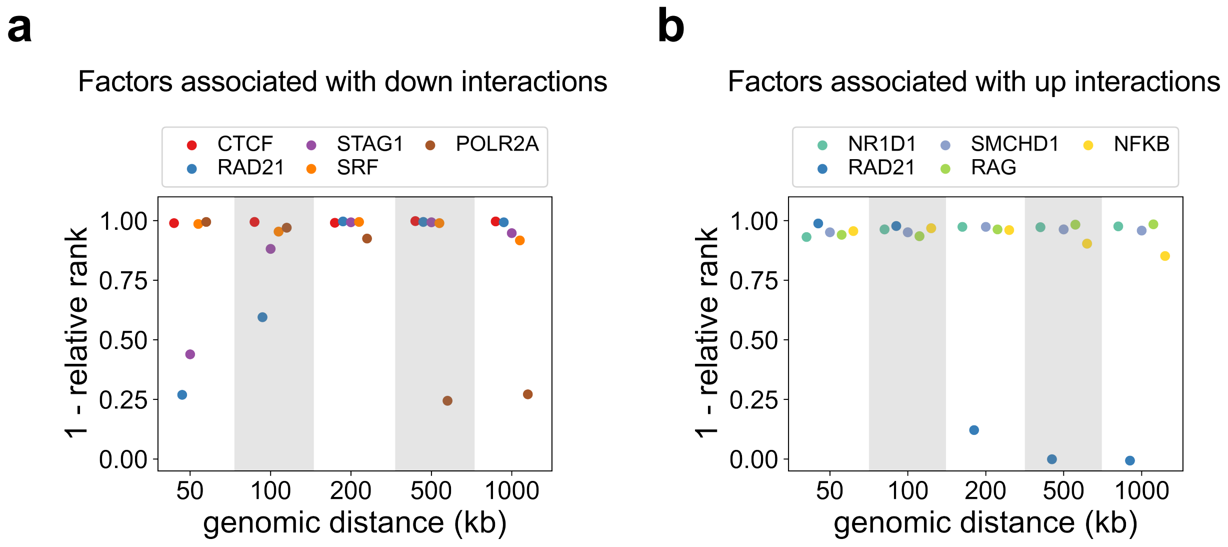

Supplementary Figure S5. BART3D results on dynamic Hi-C datasets with TR perturbation under different genomic distance settings. The data were separated for TRs associated with decreased (**a**) and increased (**b**) chromatin interactions. The 1 - relative rank of the perturbed TR in BART3D results were shown for each dataset. Higher scores correspond to higher ranked TRs.

Supplementary Table S1: Hi-C data list

Used in Figures	Species	Treatment	Treatment sample	Control sample	Replicate label
**Fig. 1e**	Mus musculus	CTCF KO	GSM2544839	GSM2544836	
**Fig. 1f**	Mus musculus	RAD21 KO	GSM3514554	GSM3514556	1
			GSM3514555	GSM3514557	2
**Fig. 1g**	Mus musculus	SMCHD1 deletion	GSM3038017	GSM3038020	1
			GSM3038018	GSM3038021	2
			GSM3038019	GSM3038022	3
**Fig. 1h,i**	Homo sapiens	differentiation	GSM2861710	GSM2861708	
**Fig. 1j**	Mus musculus	SRF overexpression	GSM2399026	GSM2399024	1
			GSM2399027	GSM2399025	2
**Fig. S3a**	Mus musculus	CTCF KO	GSM2544839	GSM2544836	
	Mus musculus	RAD21 KO	GSM3514554	GSM3514556	
	Homo sapiens	STAG2 KO	GSM3110162	GSM3110158	
	Mus musculus	SRF overexpression	GSM2399027	GSM2399025	
	Homo sapiens	triptolide	GSM3044591	GSM3044590	
**Fig. S3b**	Mus musculus	NR1D1 KO	GSM2790408	GSM2790405	
	Mus musculus	RAD21 KO	GSM3514554	GSM3514556	
	Mus musculus	SMCHD1 deletion	GSM3038018	GSM3038021	
	Mus musculus	RAG deficient	GSM2685782	GSM2418375	
	Homo sapiens	Pam3csk4 treated	GSM3111984	GSM3111983	

Supplementary Table S2: HiChIP data list

Series ID	Accession ID	Species	Cell_line	Restriction enzyme	Treatment	Capture target	Used in Figures
GSE100856	GSM2695301	Homo sapiens	lung_fibroblast	HindIII	proliferating	CTCF	Fig. S3
GSE100856	GSM2936365	Homo sapiens	umbilical_vein_endothelial	HindIII	proliferating	CTCF	Fig. S3
GSE100856	GSM2695302	Homo sapiens	lung_fibroblast	HindIII	senescent	CTCF	Fig. S3
GSE100856	GSM2936366	Homo sapiens	umbilical_vein_endothelial	HindIII	senescent	CTCF	Fig. S3
GSE101498	GSM2705060	Homo sapiens	NaÔve_T_cell	MboI	-	CTCF	Fig. S3
GSE101498	GSM2705061	Homo sapiens	NaÔve_T_cell	MboI	-	CTCF	Fig. S3
GSE105028	GSM2829030	Homo sapiens	hESC	Dpnii	control	CTCF	Fig. S3
GSE105028	GSM2829033	Homo sapiens	hESC	Dpnii	control	CTCF	Fig. S3
GSE105028	GSM2829038	Homo sapiens	hESC	Dpnii	control	CTCF	Fig. S3
GSE105028	GSM2829041	Homo sapiens	hESC	Dpnii	heat_shock	CTCF	Fig. S3
GSE105028	GSM2829042	Homo sapiens	hESC	Dpnii	heat_shock	CTCF	Fig. S3
GSE105028	GSM2829058	Homo sapiens	hESC	Dpnii	inhibit_NIPBL/heat_shock	CTCF	Fig. S3
GSE105028	GSM2829052	Homo sapiens	hESC	Dpnii	control	KLF4	Fig. S3
GSE105028	GSM2829053	Homo sapiens	hESC	Dpnii	heat_shock	KLF4	Fig. S3
GSE105028	GSM2829051	Homo sapiens	hESC	Dpnii	control	KLF4	Fig. S3
GSE105028	GSM2829048	Homo sapiens	hESC	Dpnii	control	NANOG	Fig. S3
GSE105028	GSM2829050	Homo sapiens	hESC	Dpnii	heat_shock	NANOG	Fig. S3
GSE105028	GSM2829047	Homo sapiens	hESC	Dpnii	control	NANOG	Fig. S3
GSE105028	GSM2829049	Homo sapiens	hESC	Dpnii	heat_shock	NANOG	Fig. S3
GSE105028	GSM2829043	Homo sapiens	hESC	Dpnii	control	OCT4	Fig. S3
GSE105028	GSM2829044	Homo sapiens	hESC	Dpnii	control	OCT4	Fig. S3
GSE105028	GSM2829046	Homo sapiens	hESC	Dpnii	heat_shock	OCT4	Fig. S3
GSE105028	GSM2829045	Homo sapiens	hESC	Dpnii	heat_shock	OCT4	Fig. S3
GSE105028	GSM2829016	Homo sapiens	hESC	Dpnii	control	RAD21	Fig. S3
GSE105028	GSM2829017	Homo sapiens	hESC	Dpnii	control	RAD21	Fig. S3

Supplementary Table S2: HiChIP data list

GSE105028	GSM2829018	Homo sapiens	hESC	Dpnii	control	RAD21	Fig. S3
GSE105028	GSM2829019	Homo sapiens	hESC	Dpnii	control	RAD21	Fig. S3
GSE105028	GSM2829020	Homo sapiens	hESC	Dpnii	heat_shock	RAD21	Fig. S3
GSE105028	GSM2829021	Homo sapiens	hESC	Dpnii	heat_shock	RAD21	Fig. S3
GSE105028	GSM2829022	Homo sapiens	hESC	Dpnii	heat_shock	RAD21	Fig. S3
GSE105028	GSM2829023	Homo sapiens	hESC	Dpnii	heat_shock	RAD21	Fig. S3
GSE105028	GSM2829024	Homo sapiens	hESC	Dpnii	heat_shock	RAD21	Fig. S3
GSE105028	GSM2829026	Homo sapiens	hESC	Dpnii	HSF1_kinase_inhibation	RAD21	Fig. S3
GSE108869	GSM2974085	Homo sapiens	Hela_S3	MboI	control†	CTCF	Fig. S3
GSE108869	GSM2974086	Homo sapiens	Hela_S3	MboI	control†	CTCF	Fig. S3
GSE108869	GSM2974087	Homo sapiens	Hela_S3	MboI	CTCF-s-overexpressing	CTCF	Fig. S3
GSE108869	GSM2974088	Homo sapiens	Hela_S3	MboI	CTCF-s-overexpressing	CTCF	Fig. S3
GSE116193	GSM3212925	Homo sapiens	LCL	MboI	HLA-DR3	CTCF	Fig. S3
GSE116193	GSM3212926	Homo sapiens	LCL	MboI	HLA-DR3	CTCF	Fig. S3
GSE116193	GSM3212927	Homo sapiens	LCL	MboI	HLA-DR3	CTCF	Fig. S3
GSE116193	GSM3212928	Homo sapiens	LCL	MboI	HLA-DR15	CTCF	Fig. S3
GSE116193	GSM3212929	Homo sapiens	LCL	MboI	HLA-DR15	CTCF	Fig. S3
GSE116193	GSM3212930	Homo sapiens	LCL	MboI	HLA-DR15	CTCF	Fig. S3
GSE116876	GSM3263168	Homo sapiens	MB157	MboI	Untreated	SMC1A	Fig. S3
GSE116876	GSM3263169	Homo sapiens	MB157	MboI	GSI,_Notch-off	SMC1A	Fig. S3
GSE116876	GSM3263170	Homo sapiens	MB157	MboI	GSI-washout,_Notch-on	SMC1A	Fig. S3
GSE116876	GSM3263200	Homo sapiens	HCC1599	MboI	Untreated	SMC1A	Fig. S3
GSE116876	GSM3263201	Homo sapiens	HCC1599	MboI	GSI,_Notch-off	SMC1A	Fig. S3
GSE116876	GSM3263222	Homo sapiens	Rec-1	MboI	Untreated	SMC1A	Fig. S3
GSE116876	GSM3263223	Homo sapiens	Rec-1	MboI	GSI,_Notch-off	SMC1A	Fig. S3

Supplementary Table S2: HiChIP data list

GSE119997	GSM3397790	Homo sapiens	hESC	MboI	P63KO_w/_morphogen	SMC1A	Fig. S3
GSE119997	GSM3397781	Homo sapiens	hESC	MboI	WT	SMC1A	Fig. S3
GSE119997	GSM3397783	Homo sapiens	hESC	MboI	express_P63_no_morphogen	SMC1A	Fig. S3
GSE119997	GSM3397784	Homo sapiens	hESC	MboI	express_P63_no_morphogen	SMC1A	Fig. S3
GSE119997	GSM3397785	Homo sapiens	hESC	MboI	express_P63_no_morphogen	SMC1A	Fig. S3
GSE119997	GSM3397786	Homo sapiens	hESC	MboI	express_P63_w/_morphogen	SMC1A	Fig. S3
GSE119997	GSM3397787	Homo sapiens	hESC	MboI	express_P63_w/_morphogen	SMC1A	Fig. S3
GSE119997	GSM3397788	Homo sapiens	hESC	MboI	express_P63_w/_morphogen	SMC1A	Fig. S3
GSE119997	GSM3397789	Homo sapiens	hESC	MboI	P63KO_w/_morphogen	SMC1A	Fig. S3
GSE119997	GSM3397791	Homo sapiens	hESC	MboI	P63KO_w/_morphogen	SMC1A	Fig. S3
GSE119997	GSM3397780	Homo sapiens	hESC	MboI	WT	SMC1A	Fig. S3
GSE119997	GSM3397782	Homo sapiens	hESC	MboI	WT	SMC1A	Fig. S3
GSE80820	GSM2238512	Mus musculus	v6.5_mESC	MboI	-	OCT4	Fig. S3
GSE80820	GSM2238504	Mus musculus	v6.5_mESC	MboI	-	OCT4	Fig. S3
GSE80820	GSM2238508	Mus musculus	v6.5_mESC	MboI	-	OCT4	Fig. S3
GSE80820	GSM2238510	Mus musculus	v6.5_mESC	MboI	-	OCT4	Fig. S3
GSE80820	GSM2138328	Mus musculus	v6.5_mESC	MboI	-	SMC1A	Fig. S3
GSE80820	GSM2138329	Mus musculus	v6.5_mESC	MboI	-	SMC1A	Fig. S3
GSE80820	GSM2138326	Homo sapiens	GM12878	MboI	-	SMC1A	Fig. S3
GSE80820	GSM2138327	Homo sapiens	GM12878	MboI	-	SMC1A	Fig. S3
GSE80820	GSM2138330	Mus musculus	v6.5_mESC	MboI	-	SMC1A	Fig. S3
GSE80820	GSM2138331	Mus musculus	v6.5_mESC	MboI	-	SMC1A	Fig. S3
GSE80820	GSM2138332	Mus musculus	v6.5_mESC	MboI	1m_cells	SMC1A	Fig. S3
GSE80820	GSM2138333	Mus musculus	v6.5_mESC	MboI	1m_cells	SMC1A	Fig. S3
GSE80820	GSM2138335	Mus musculus	v6.5_mESC	MboI	5m_cells	SMC1A	Fig. S3
GSE80820	GSM2138336	Mus musculus	v6.5_mESC	MboI	10m_cells	SMC1A	Fig. S3

Supplementary Table S2: HiChIP data list

GSE80820	GSM2138337	Mus musculus	v6.5_mESC	MboI	10m_cells	SMC1A	Fig. S3
GSE80820	GSM2138324	Homo sapiens	GM12878	MboI	-	SMC1A	Fig. S3
GSE80820	GSM2138325	Homo sapiens	GM12878	MboI	-	SMC1A	Fig. S3
GSE80820	GSM2138334	Mus musculus	v6.5_mESC	MboI	5m_cells	SMC1A	Fig. S3
GSE92881	GSM3049595	Homo sapiens	HCT-116	MboI	-	SMC1A	Fig. S3
GSE99521	GSM2774001	Homo sapiens	Jurkat	MboI	-	YY1	Fig. S3
GSE99521	GSM2774000	Homo sapiens	HCT116	MboI	-	YY1	Fig. S3
GSE99521	GSM2774002	Homo sapiens	K562	MboI	-	YY1	Fig. S3

Supplementary Table S3: ChIP-seq data list

GSM ID	Species	ChIP factor	Used in Figures
GSM2538390	Mus musculus	CTCF	Fig. 1b
GSM1187181	Mus musculus	RAD21	Fig. 1c
GSM1604019	Mus musculus	SMCHD1	Fig. 1d

Supplementary Table S4

a

Rad21 KO

	T1-C1	T1-C2	T2-C1	T2-C2	combined
**T1-C1**	1	0.93	0.85	0.81	0.90
**T1-C2**		1	0.81	0.86	0.90
**T2-C1**			1	0.94	0.90
**T2-C2**				1	0.90
**combined**					1

b

Smchd1 KO

	T1-C1	T1-C2	T1-C3	T2-C1	T2-C2	T2-C3	T3-C1	T3-C2	T3-C3	combined
**T1-C1**	1	0.89	0.88	0.90	0.20	0.17	0.86	0.16	0.15	0.78
**T1-C2**		1	0.90	0.17	0.84	0.25	0.23	0.76	0.28	0.78
**T1-C3**			1	0.14	0.26	0.89	0.13	0.20	0.83	0.78
**T2-C1**				1	0.90	0.89	0.85	0.15	0.15	0.78
**T2-C2**					1	0.90	0.23	0.73	0.26	0.78
**T2-C3**						1	0.13	0.17	0.82	0.78
**T3-C1**							1	0.91	0.91	0.84
**T3-C2**								1	0.91	0.83
**T3-C3**									1	0.84
**combined**										1

c

Srf over expression

	rep1-1	rep1-2	rep2-1	rep2-2	combined
**T1-C1**	1	0.93	0.95	0.73	0.83
**T1-C2**		1	0.71	0.94	0.83
**T2-C1**			1	0.93	0.83
**T2-C2**				1	0.83
**combined**					1

d

Rad21 KO
decreased interactions

	Rank of RAD21
**T1-C1**	2
**T1-C2**	2
**T2-C1**	2
**T2-C2**	2
**combined**	2

e

Smchd1 KO
increased interactions

	Rank of SMCHD1
**T1-C1**	12
**T1-C2**	20
**T1-C3**	5
**T2-C1**	7
**T2-C2**	9
**T2-C3**	9
**T3-C1**	33
**T3-C2**	36
**T3-C3**	30
**combined**	11

f

Srf over expression
increased interactions

	Rank of NANOG
**T1-C1**	14
**T1-C2**	19
**T2-C1**	20
**T2-C2**	11
**combined**	15

Supplementary Table S4. Comparison of BART3D results between individual replicates and replicate combined data. (a-c) Pearson correlation coefficient of pairwise comparisons of BART3D derived DCI profiles between individual replicates and replicate combined data for Rad21 KO **(a)**, Smchd1 KO **(b)** and Srf over expression **(c)**. **(d-f)** Rank of relevant TR in BART3D results from using individual replicates and replicate combined data for Rad21 KO **(d)**, Smchd1 KO **(e)** and Srf over expression **(f)**. In all panels, Ti-Cj refers to BART3D results from using treatment sample i over control sample j, combined: BART3D results from using all treatment replicates over all control replicates.

Supplementary Table S5

a

Correlation coefficient with read coverage	DCI	Adjusted DCI
Ctcf KO	0.09	0
Rad21 KO	0.14	0
Smchd1 KO	0.08	0
Srf OE	0.11	0
EP vs HSPC	0.24	0

b Ctcf KO decreased interactions

	**CTCF rank**
DCI	5
Adjusted DCI	4

c Rad21 KO decreased interactions

	**RAD21 rank**
DCI	2
Adjusted DCI	2

d Smchd1 KO increased interactions

	**SMCHD1 rank**
DCI	11
Adjusted DCI	9

e EP over HSPC increased interactions

	**GATA1 rank**
DCI	1
Adjusted DCI	1

f EP over HSPC decreased interactions

	**GATA3 rank**
DCI	1
Adjusted DCI	1

g Srf over expression increased interactions

	**NANOG rank**
DCI	15
Adjusted DCI	14

Supplementary Table S5. Optional adjustment of DCI by locus read coverage. (a) Pearson correlation coefficient between locus read coverage change (log2 fold change of normalized reads) of Hi-C data and the original DCI or the coverage-adjusted DCI. **(b-g)** Ranks of relevant TR in BART3D results using original DCI or coverage-adjusted DCI derived from Hi-C data in Ctcf KO **(b)**, Rad21 KO **(c)**, Smchd1 KO **(d)**, EP over HSPC **(e,f)**, and Srf over expression **(g)**.

Supplementary Table S6

	Selfish	diffHic	BART3D
Rank of CTCF in CTCF KO decreased interaction	1/565	N.A.	5/565
Rank of RAD21 in RAD21 KO decreased interaction	5/565	N.A.	2/565
Rank of SMCHD1 in SMCHD1 KO increased interaction	396/565	394/565	11/565

Supplementary Table S6. Comparison of BART3D TR prediction with Selfish and diffHic. Rank of relevant TR on decreased or increased chromatin interactions from Selfish, diffHic and BART3D for three Hi-C datasets. For Selfish and diffHic, BART analysis was applied to anchor regions of identified loops exhibiting increased/decreased chromatin interaction. N.A., no differential loop was generated from diffHic.
